# Supplementary material for: Design, Synthesis, and Biological Evaluation of N-Acylhydrazones and Their Activity Against Leishmania amazonensis Promastigotes
Source: Microorganisms. 2025 Jul 2;13(7):1563. doi: 10.3390/microorganisms13071563 (PMC12298860; doi:10.3390/microorganisms13071563)
Supplement: Supplementary file 1 [file microorganisms-13-01563-s001.zip › HPLC amostras-20241114T144635Z-001.zip!/HPLC amostras/HPLC composto 12 - PMM 102 - 17.pdf]

# Farmanguinhos - Fiocruz
# Serviço de Métodos Analíticos

PMM 102 - 17.lcd
2018 - Sintese 1 - João Mafra - 2-5/2-212-4 - PMM 102 - 17.lcd
25/06/2018, 12:02:55

| | | | |
|---|---|---|---|
| Sample Name | : PMM 102 - 17 | | |
| Sample ID | : 118739 | | |
| Sample Full Path | : 2018 - Sintese 1 - João Mafra - 2-5/2-212-4 - PMM 102 - 17.lcd | | |
| Data Filename | : PMM 102 - 17.lcd | | |
| Method Filename | : Nitroimidazol.lcm | | |
| Batch File | : 2018 - Sintese 1 - João Mafra - CLAE-10 - CLAE 10_2018 _0619.lcb | | |
| Vial # | : 1-75 | Sample Type | : Unknown |
| Injection Volume | : 10 uL | | |
| Date Acquired | : 25/06/2018 12:03:24 | Acquired by | : Marcio Soares |
| Date Processed | : 27/06/2018 09:18:08 | Processed by | : Marcio Soares |
| Data Comment | : Método: Nitroimidazol | | |

Preparo da Amostra: Cerca de 1 mg de amostra foram adicionados 300 µL de DMSO, após completa solubilização, a solução foi avolumada com metanol, obtendo um volume final de 4 mL, agitada em vortex. Posteriormente filtrada em membrana PVDF de 0,22µm e injetada. Massa informada pelo solicitante.

## Method

<<Header>>

<<Pump>>
Total Flow : 1,0000 mL/min
Pump A : LC-20ADXR

<<Mobile Phase Name>>
Pump A Mobile Phase A : Água
Pump A Mobile Phase C : Acetonitrila

<<LC Time Program>>

| Time | Module | Command | Value | C |
|---|---|---|---|---|
| 0,01 | Pumps | Solvent C Conc. | 20 | |
| 0,01 | Pumps | Total Pump A Flow | 1 | |
| 5,00 | Pumps | Solvent C Conc. | 20 | |
| 22,00 | Pumps | Solvent C Conc. | 90 | |
| 25,00 | Pumps | Solvent C Conc. | 90 | |
| 33,00 | Pumps | Solvent C Conc. | 20 | |
| 39,00 | Pumps | Solvent C Conc. | 20 | |
| 39,01 | Controller | Stop | | |

<<Data Acquisition>>
LC Stop Time : 39,01 min

<<Oven>>
Oven Temperature : 30 C

## Instrumentation

<<Instrument>>

<<Pump A>>
Pump A Type : LC-20ADXR

<<System Controller>>
SCL Type : CBM-20A

<<Autosampler>>
Autosampler : SIL-30AC

<<Oven>>
Oven Type : CTO-20AC

<<Column>>
Column Name : Supelcosil LC- 8 - 5 µm
Column ID : CL 0173
Length : 150 mm
Internal Diameter : 4,6 mm
Max Number of Injections : 9999
Expiration Date : 28/04/2015
Max Temperature : 80,0 C
Installation Date : 28/04/2030

<<PDA>>
Detector Name : PDA
Detector Type : SPD-M20A

<<Degasser>>
Degasser : DGU-20A5R

# Farmanguinhos - Fiocruz
# Serviço de Métodos Analíticos

PMM 102 - 17.lcd
2018 - Sintese 1 - João Mafra - 2-5/2-212-4 - PMM 102 - 17.lcd
25/06/2018, 12:02:55

Chromatogram

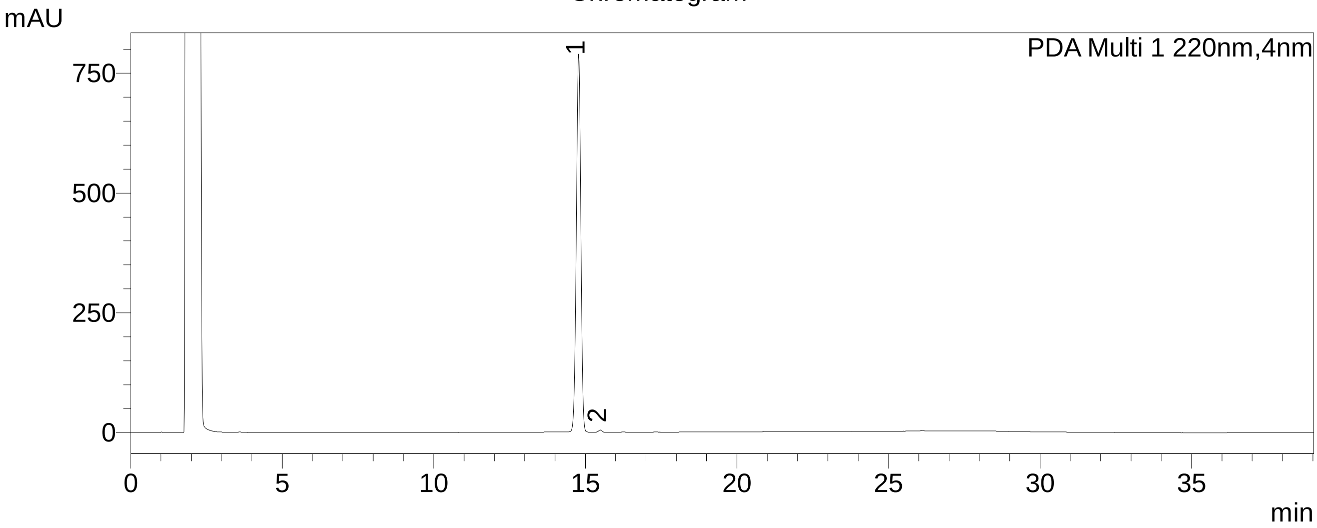


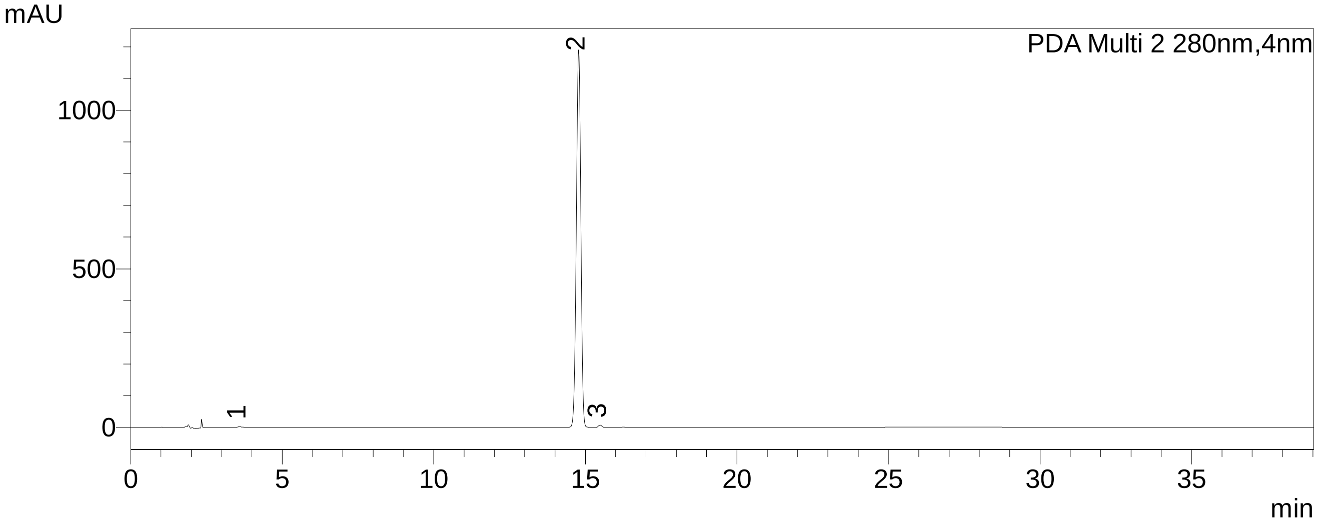


Peak Table

PDA Ch1 220nm

| Peak# | Ret. Time | Name | Area | Area% | Theoretical Plates/meter(USP) | Tailing Factor | Resolution(USP) | Capacity Factor(k') |
|---|---|---|---|---|---|---|---|---|
| 1 | 14,78 | | 8157002 | 99,6 | 283668 | 0,970 | -- | -- |
| 2 | 15,49 | | 35313 | 0,4 | 511703 | 0,999 | 2,764 | 0,048 |
| Total | | | 8192315 | 100,0 | | | | |

PDA Ch2 280nm

| Peak# | Ret. Time | Name | Area | Area% | Theoretical Plates/meter(USP) | Tailing Factor | Resolution(USP) | Capacity Factor(k') |
|---|---|---|---|---|---|---|---|---|
| 1 | 3,59 | | 15099 | 0,1 | 24173 | 1,163 | -- | -- |
| 2 | 14,78 | | 12385112 | 99,4 | 281647 | 0,970 | 42,515 | 3,114 |
| 3 | 15,49 | | 59586 | 0,5 | 508029 | 0,990 | 2,756 | 3,310 |
| Total | | | 12459797 | 100,0 | | | | |
